# Ten Years of *PLoS^‡^ Computational Biology:* A Decade of Appreciation and Innovation

**DOI:** 10.1371/journal.pcbi.1004317

**Published:** 2015-06-24

**Authors:** Philip E. Bourne, Steven E. Brenner, Michael B. Eisen

**Affiliations:** 1 Office of the Director, National Institutes of Health, Bethesda, Maryland, United States of America; 2 Department of Plant and Microbial Biology, University of California, Berkeley, Berkeley, California, United States of America; 3 Department of Molecular and Cell Biology, University of California, Berkeley, Berkeley, California, United States of America

‡ *Since the founding of* PLoS Computational Biology, *our publisher, the Public Library of Science, has evolved its own gloss from the piquantly camelesque “PLoS” to the modern “PLOS,” matching its equally emphatic journal ONE. As we review the journal, it seemed appropriate to hearken to its uncertain beginnings, embodied even in its quirky typography*.

Ten years ago, we founded *PLoS Computational Biology* to advance the science of computational biology and its practitioners. Our goals were appreciation and innovation. At the remove of no longer being active in day-to-day activities of the journal, it is wonderful to look back over the first ten years of the journal. Here we reflect on the journal’s evolution and offer a few thoughts for the next ten years.

The journal began at a propitious time, as computational biology engaged markedly more researchers and as scientific publication was beginning a tectonic shift. Many researchers noted a serious gap in the publishing firmament: there was no respected venue that focused on biological discovery made by computational methodology. This lack of appreciation had implications at many levels. At pre-eminent journals, computational biology research was often was not reviewed by those with expertise to evaluate its technical content and significance, leading to delays and rejections. The alternative of publishing at the existing specialist journals often led to publications in venues that were not appreciated by the most appropriate audience. This meant that the work was not seen by many suitable readers, and that computational biologists had greater challenges in their career development.

We are pleased that *PLoS Computational Biology* is now widely appreciated as a treasury of first-class scientific work, offering a respected venue for publication of work fundamental to our field. For researchers at all tiers, but especially trainees, publication venue is too often taken as a proxy for the significance of their research studies. Such individuals have no time for their papers to accrue citations or become well recognized before their achievements are assessed for career advancement. We hope that those who have published in *PLoS Computational Biology* have met with success comparable to the burnishing their outstanding work has provided for the journal’s reputation.

Even more important, we have been gratified to see researchers in our field have their work treated with respect and insight by reviewers and editors who understand the significance of their research and its approaches. Certainly, in a journal that rejects two-thirds of the submissions, not all prospective authors are happy, and undoubtedly there have been some significant oversights. However, on the whole, we believe that *PLoS Computational Biology* has succeeded in providing computational biology researchers consistently informed and thoughtful reviews.

In order to meet the needs of our field’s researchers, our intent was to establish a computational journal that was read by experimentalists and computational researchers alike, and would drive biologists’ interest in the application of computational techniques. Another early motivator was also not to encroach on existing bioinformatics journals, hence, the niche of computation requiring new biological insights. To this end, *PLoS Computational Biology* initially had a scope statement that emphasized the need for biological outcomes. However, as both our journal and bioinformatics journals became more established, the editorial board increasingly felt we should expand the remit to engage pure methods of the greatest significance. The expansion of authors and readers has been clearly enhanced by embracing methods papers.

A pleasant and unexpected surprise was the scientific scope of papers submitted to the journal. For example, we never envisaged that computational neuroscience would become such a vibrant part of the journal. In retrospect, this would seem to be the result of a combination of lack of suitable venues publishing such papers and the hard work of the early neuroscience editors. The scope has continued to expand even as the number of journals competing has multiplied. An imminent challenge will likely be understanding the relationship with biomedical informatics as the line between basic and clinical research blurs in a translational world. A broader challenge will arise as biology becomes ever more a data science: what should the unique role of the journal be as nearly all biologists become computational biologists?

The journal began at a time when open access was still considered by most scientists at best a novelty and at worst a business model doomed to failure. Against this backdrop, many computational biologists rallied behind the cause since they were already accustomed to open and free data and software. A particularly important leap was made by the International Society for Computational Biology (ISCB), which was a key founding partner. Not only did ISCB offer a community supporting the journal, but in doing so, they abandoned the very substantial revenues they had previously garnered from partnership from a journal that was then closed-access. Valuing access over profit is to be highly commended.

In the ten interceding years, open access has evolved and is now a mainstream publishing option, which has undoubtedly increased access to scientific discourse. Importantly, *PLoS Computational Biology* has the most elevated tier of open access, using a CC-By license. Amongst other benefits, for our community, this offers machine access to the corpus for knowledge discovery, an undertaking that remains at a nascent stage. Let us hope that such value is commonplace and plain for all to see before the next ten-year anniversary.

The front matter has been a wonderful sandbox in which to experiment with scholarly communication. Not all such experiments have been successful, but some clearly have and can be attributed to both filling a need (not necessarily obvious at the outset) and folks who believed in the experiment and who put in many hours of hard work on behalf of the community. The Education section and the Ten Simple Rules professional development series have blossomed beautifully. The jury is still out on the success of other experiments, but they all speak to doing things differently, which has been a hallmark of the journal since day one.

The science of computational biology and the dissemination of that science have changed over the past ten years, and the journal has been there to document and enable those changes, and more disruption will follow. The next ten years will see a further broadening of the scope of what is covered by the field of computational biology, particularly as it relates to the generation and use of a broader range of data types by all investigators, with increased emphasis on the use of clinical data. We hope that more of the research life cycle will be shared and captured as part of a publication. If dreams come true, a journal paper will offer a persistent mechanism for further data exploration and be capable of offering a platform for follow-on sets of experiments. We are sure your community journal will be ready for whatever innovations you can bring.

It was a privilege for us to work with so many talented authors, reviewers, editors, and staff—and an honor to see our initial efforts taken to such wonderful levels. All have contributed both appreciation and innovation and, in doing so, helped create a community larger than the journal itself. All of their efforts have truly elevated the field and supported us.

